# CRISPR screens in the context of immune selection identify *CHD1* and *MAP3K7* as mediators of cancer immunotherapy resistance

**DOI:** 10.1016/j.xcrm.2025.102565

**Published:** 2026-01-20

**Authors:** Alex Watterson, Gabriele Picco, Vivien Veninga, Youhani Samarakoon, Chiara M. Cattaneo, Sara F. Vieira, Emre Karakoc, Shriram Bhosle, Thomas W. Battaglia, Sarah Consonni, Timotheus Y.F. Halim, Emile E. Voest, Mathew J. Garnett, Matthew A. Coelho

**Affiliations:** 1 Cancer, Ageing and Somatic Mutation Programme, Wellcome Sanger Institute, Hinxton, Cambridgeshire, UK; 2 Open Targets, Cambridgeshire, UK; 3 Department of Immunology and Molecular Oncology, Netherlands Cancer Institute, Amsterdam, the Netherlands; 4 Oncode Institute, Utrecht, the Netherlands; 5 Cancer Research UK, Cambridge Institute, University of Cambridge, Cambridge, UK

**Keywords:** CHD1, MAP3K7, TAK1, CRISPR-Cas9, co-culture screen, T cells, cancer immunotherapy, resistance, IFN-γ, immmune checkpoint blockade

## Abstract

Cancer immunotherapy is only effective in a subset of patients, highlighting the need for effective biomarkers and combination therapies. Here, we systematically identify genetic determinants of cancer cell sensitivity to anti-tumor immunity by performing whole-genome CRISPR-Cas9 knockout screens in autologous tumoroid-T cell co-cultures, isogenic cancer cell models deficient in interferon signaling, and in the context of four cytokines. We discover that loss of *CHD1* and *MAP3K7* (encoding TAK1) potentiates the transcriptional response to IFN-γ, thereby creating an acquired vulnerability by sensitizing cancer cells to tumor-reactive T cells. Immune checkpoint blockade is more effective in a syngeneic mouse model of melanoma deficient in *Chd1* and *Map3k7* and is associated with elevated intra-tumoral CD8^+^ T cell numbers and activation. *CHD1* and *MAP3K7* are recurrently mutated in cancer, and reduced expression in tumors correlates with response to immune checkpoint inhibitors in patients, nominating these genes as potential biomarkers of immunotherapy response.

## Highlights

• CRISPR-Cas9 co-culture screens with patient-derived tumoroids and autologous T cells
• Large-scale map of cytokine-induced cancer dependencies
• *CHD1* and *MAP3K7* (TAK1) loss enhances sensitivity to IFN-γ
• Low tumor expression of *CHD1*/*MAP3K7* is linked to improved ICB response in mice and patients.

## Graphical abstract

**Figure:**
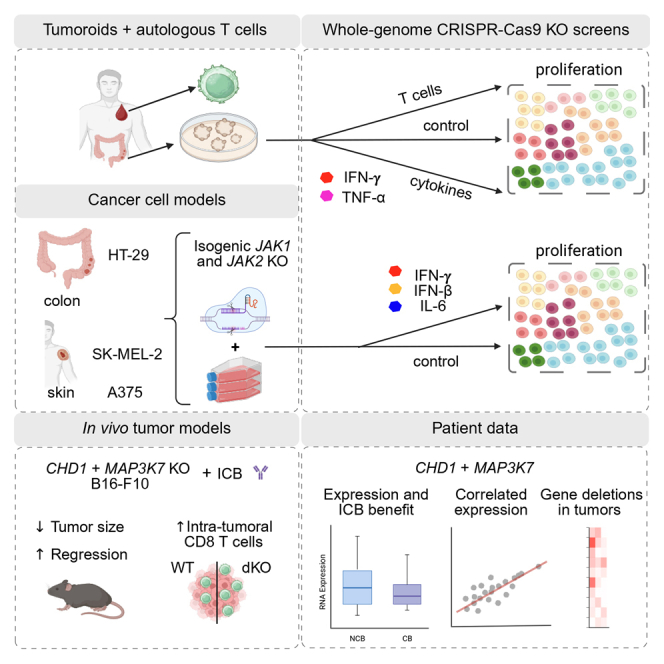

## Teaser

Immunotherapies only provide benefit for a subset of individuals with cancer, highlighting the need for biomarkers of response. Using whole-genome CRISPR-Cas9 screens, Watterson et al. map genetic dependencies in the context of four cytokines and tumor-reactive T cells, identifying *CHD1* and *MAP3K7* as mediators of resistance to IFN-γ-induced cancer cell death.

## Introduction

Immune checkpoint blockade (ICB) is an effective treatment for many cancer types, such as melanoma and cancers with microsatellite instability (MSI);^1^ however, response rates in other solid tumors are below 35%.^2^ Understanding the genetic determinants of ICB response would help to identify effective combination therapies^3^ and stratify individuals who benefit from treatment, sparing others from treatment-related toxicities.^4^

For patients who initially respond to ICB, acquired resistance can emerge through various mechanisms,^5^ including *HLA* or *B2M* mutation,^6^^,^^7^ or disabling the IFN-γ pathway through mutations in *JAK1*/2,^8^^,^^9^ highlighting the importance of tumor-cell-intrinsic signaling in immune evasion. CRISPR-Cas9 knockout (KO) screens have identified genetic modulators of IFN-γ response and sensitivity to T cells.^10^^,^^11^^,^^12^ However, these studies typically cannot discriminate the independent effects of different cytokines or deconvolute pathway-specific biology,^13^^,^^14^ rely on overexpression of artificial antigens,^15^ or focus on mouse model systems that use highly immunogenic cell lines.^13^^,^^14^

Loss of tumor suppressor genes drives cancer progression and therapy resistance^16^ but can also have important non-cell-autonomous effects^17^ and result in acquired vulnerabilities.^18^^,^^19^ *CHD1* (chromodomain helicase DNA binding protein 1) and *MAP3K7* (encoding transforming growth factor β-activated kinase 1; TAK1) have been proposed as tumor suppressor genes^20^^,^^21^^,^^22^ due to their recurrent mutation in prostate cancers^23^^,^^24^ and loss at lower frequencies in other cancer types.^25^^,^^26^ In prostate cancer, co-deletion is associated with aggressive disease and therapy resistance.^27^^,^^28^^,^^29^ Loss of *CHD1*, a chromatin-remodeling enzyme,^30^ is associated with extensive changes in the tumor microenvironment (TME) in mice through reduced NF-*κ*B signaling.^31^^,^^32^ *MAP3K7* is found within a commonly deleted genomic region in prostate cancer (chromosome 6q15)^29^ and encodes a kinase involved in activating NF-*κ*B and TGF-β signaling.^33^^,^^34^^,^^35^ The effects of *MAP3K7* loss on the TME and ICB response remain largely unexplored.

Here, we investigate the genetic landscape of sensitivity to autologous human T cells and cytokines found in the TME in four cancer cell models, generating a genome-scale map of context-specific dependencies. Integrative analysis reveals that *CHD1* and *MAP3K7* loss additively sensitizes cancer cells to IFN-γ and anti-tumor T cells and that reduced expression in tumors is associated with patient response to ICB.

## Results

### Functional genomics identifies genetic modulators of cytokine response

**Figure 1:**
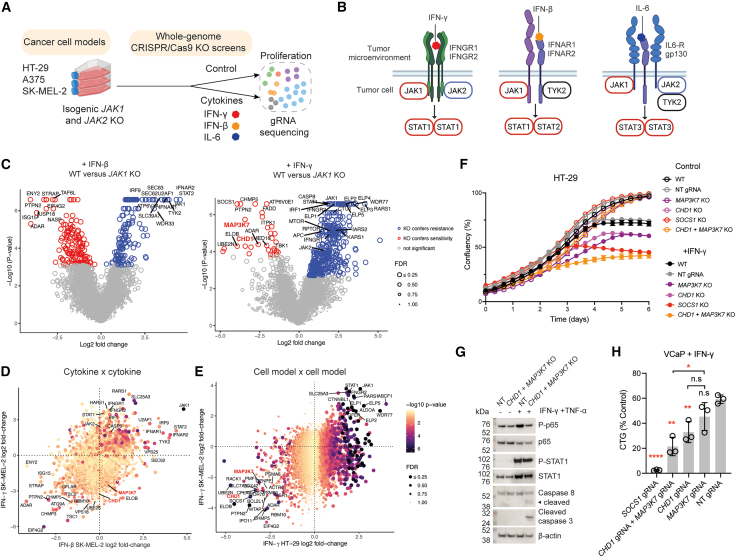
Whole-genome CRISPR screens define the genetic determinants of cytokine sensitivity in cancer cells (A) Overview of CRISPR-Cas9 screens to identify modulators of IFN-γ, IFN-β, and IL-6 cytokine responses in three cancer cell models. (B) Overview of cytokine signaling pathways and differential dependencies on JAK1 and JAK2. (C) Gene-level volcano plots of CRISPR-Cas9 KO screens comparing wild-type (WT) and *JAK1* KO SK-MEL-2 cells treated with IFN-β (400 U/mL, left panel) or HT-29 cells treated with IFN-γ (500 U/mL, right panel). Data represent the average of two independent screens, and significant hits are highlighted (*P*-adjusted < 0.05, false discovery rate [FDR] < 0.1). (D) Shared and private modulators of IFN-γ and IFN-β responses in SK-MEL-2 cells. Scatterplots compare the log2 fold-change of WT control versus IFN-γ- or IFN-β-treated *JAK1* KO clones. Data represent the average of two independent screens and are representative of two independent *JAK1* KO clones. FDR and *p*-values are indicated for the cytokine with the largest effect size. (E) CRISPR screens identify genes conferring resistance or sensitivity to IFN-γ in SK-MEL-2 and HT-29 cancer cell models. Scatterplots compare log 2-fold-change values for WT control versus IFN-γ-treated conditions. *P*-adjusted values and FDR values for HT-29 screens are indicated. Data represent the average of two independent screens. (F) *CHD1* and *MAP3K7* KO additively sensitize cancer cells to IFN-γ. Cell growth Incucyte curves from *CHD1, MAP3K7, CHD1* and *MAP3K7* dKO, and *SOCS1* KO HT-29 ± IFN-γ (500 U/mL). Data represent the mean ± SD of three biological replicates and are representative of two independent experiments. Data are representative of two different gRNAs targeting *CHD1* or *MAP3K7*. (G) *CHD1* and *MAP3K7* KO prime cancer cells for apoptosis in response to IFN-γ and TNF-*α*. Western blotting of HT-29 NT gRNA control cells or *CHD1* and *MAP3K7* dKO cells ± IFN-γ (500 U/mL) and TNF-*α* (100 ng/mL) for 8 h. Data are representative of two independent experiments. (H) Sensitivity of *CHD1* and *MAP3K7* KO prostate cancer cells to IFN-γ. VCaP cells were grown ± IFN-γ (500 U/mL), and cell viability was measured using Cell Titer-Glo (CTG). Data were normalized to untreated controls and represent the mean ± SD of three independent experiments performed in technical triplicate. Two-tailed, unpaired Student’s *t* test comparing NT gRNA to KO conditions, or single KO conditions to dKO as indicated by brackets; ∗*P* < 0.05; ∗∗*P* < 0.01; ∗∗∗∗*P* < 0.0001; not significant, n.s; NT, non-targeting.

To systematically investigate the genetic determinants of cytokine response in cancer cells, we performed whole-genome CRISPR-Cas9 KO screens (STAR Methods^36^) in the presence of cytokines found in the TME (Figure 1A). First, we tested IFN-γ, IFN-β, and IL-6, which affect anti-tumor immunity and depend on JAK1 or JAK2 signaling^37^ (Figure 1A). We selected cell models from cancer types in which immunotherapy is used clinically, such as melanoma^38^ (A375 and SK-MEL-2) and colorectal cancer (CRC)^39^ (HT-29) and generated two independent isogenic *JAK1* or *JAK2* KO clones for each cell model (Figures S1A–S1D) to investigate JAK1- or JAK2-specific signaling dependencies (Figure 1B). The IFN-γ and IL-6 receptors engage both JAK1 and JAK2,^40^^,^^41^ whereas the IFN-β receptor engages JAK1 and TYK2,^41^ consistent with disparate cytotoxic responses to IFN-β in *JAK1* and *JAK2* KO SK-MEL-2 clones (Figures 1B and S1E). Replicate correlation for independent CRISPR-Cas9 KO screens in the absence of cytokine stimulation was high, and essential genes were depleted,^42^ verifying screen quality (*r* = 0.82–0.91; Figures S1F–S1H; Table S1). Variability arose in the IL-6 screen conditions due to minimal impact on cell growth despite stimulation of signaling (Figures S1A–S1D). SK-MEL-2 *JAK1* KO screens highlighted known IFN-β biology, including sensitizer (*ADAR*,^43^ *PTPN2*^10^ KO) and resistance hits (*TYK2*, *STAT2*, and *IFNAR1/2* KO). These were absent in *JAK2* KO screens (Figure S2A), consistent with the contribution of JAK1, but not JAK2, in IFN-β signaling.

To investigate variation in genetic mediators of response between different cytokines, we compared dependencies in IFN-γ and IFN-β screens (Figures 1C and 1D). *ADAR* and *PTNP2* KO were sensitizing, and *JAK1* KO conferred resistance to both cytokines, whereas KO of the genes encoding the receptors for IFN-γ or IFN-β (*IFNGR1/2* and *IFNAR1/2*) provided cytokine-specific resistance. Genes involved in vesicular trafficking and mTOR signaling were sensitizing hits in both cytokine contexts, including *TSC1*, *TSC2*, *CHMP3*, and *CHMP5* (Figure S2B).

Comparison of IFN-γ response-modulating genes across cancer cell models identified KO of *JAK1*, *STAT1*, *IRF1*, and *CASP8* as conferring resistance in all three cell models (Figure S2C). KO of *CFLAR*^44^ was universally sensitizing to IFN-γ, consistent with a caspase 8-mediated mechanism of apoptosis.^45^ *MTOR* and *RPTOR* were shared resistance hits, implicating cell-intrinsic mTOR signaling in cancer cell IFN-γ response. Other shared resistance hits pertained to tRNA processing (e.g., *RARS1*) and amino acid transport (e.g., *SLC25A3*) (Figure S2D). These genes are thought to relate to mTOR signaling through modulation of cellular amino acid levels.^46^^,^^47^ IFN-γ-sensitizing hits shared across three cancer cell models (*n* = 8) included *TSC1*, *SLC7A5*, *RAB7A*, and *CHMP5*, relating to mTOR and vesicular trafficking. Moreover, gene ontology and pathway analysis of shared sensitizing and resistance hits across cancer cell models revealed significant enrichment in tRNA processing and translation (Figure S2E).

Through screening 15 cell models (wild type [WT], *JAK1* KO, and *JAK2* KO) treated with three cytokines, we mapped context-specific genetic dependencies from 138 whole-genome CRISPR-Cas9 KO screen samples (Table S2). Collectively, these data outline both conserved and context-specific genetic modulators of cytokine signaling in cancer cells and highlight the importance of the mTOR pathway in mediating cancer cell-intrinsic cytokine responses.^13^^,^^41^^,^^48^

### *CHD1* and *MAP3K7* loss sensitizes cancer cells to IFN-γ

To discover genes that could serve as biomarkers of cancer response to inflammatory cytokines using this combined dataset, we focused on IFN-γ-sensitizing hits with minimal impact on cell fitness in the absence of IFN-γ, such as *PTPN2* and *SOCS1* (Figure S2F). Two such hits were *CHD1* and *MAP3K7* (Figures 1E, S2F, S2G, and S3A). *CHD1* and *MAP3K7* are co-deleted in prostate cancers and are on separate chromosomes,^28^ implying a cooperative mechanism. Consistently, analysis of 51 studies from The Cancer Genome Atlas^49^ (TCGA) and literature^27^^,^^50^^,^^51^^,^^52^^,^^53^^,^^54^^,^^55^^,^^56^^,^^57^^,^^58^^,^^59^^,^^60^^,^^61^^,^^62^^,^^63^^,^^64^^,^^65^^,^^66^^,^^67^^,^^68^^,^^69^^,^^70^ highlighted prostate cancer as having the highest incidence of loss; 1.2%–16.9% of tumor samples had deletion of one gene, and 0.7%–3.9% had co-deletions of *CHD1* and *MAP3K7* (Figure S3B). Other cancer types with recurrent co-deletions included thyroid cancer (4.7%), melanoma (0.2–3.7%), and meningioma (0.8%).

To validate our findings from genome-wide CRISPR-Cas9 KO screens, we performed arrayed KO experiments. *CHD1* or *MAP3K7* KO (Figure S3C) did not alter cell proliferation rate in HT-29 but sensitized cells to IFN-γ (Figure 1F). To model co-deletion, we knocked out both genes together (double KO, dKO). This led to more profound sensitization to IFN-γ than either single-gene KO, indicating an additive effect (Figure 1F). Single KO and dKO HT-29 cells had similar levels of JAK-STAT activation to WT cells in response to IFN-γ or IFN-γ and TNF-*α* (P-STAT1; Figures 1G and S3D) but exhibited reduced NF-*κ*B activity (P-p65) and increased induction of apoptosis (cleaved caspase-3 and -8; Figures 1G and S3D). Takinib, a TAK1 inhibitor,^71^ phenocopied *MAP3K7* loss on IFN-γ sensitivity, specifically in the context of *CHD1* KO (Figure S3E), although not to the same extent as *MAP3K7* KO, suggesting incomplete inhibition. Furthermore, in the prostate cancer model VCaP, dKO cells had increased sensitivity to IFN-γ (Figures 1H and S3F) and were significantly more sensitive than the *MAP3K7* single KO but not the *CHD1* single KO. Taken together, these data highlight *CHD1* and *MAP3K7* loss as a cytokine-dependent vulnerability in cancer cells.

### Autologous tumoroid-T cell co-culture CRISPR screens identify modulators of cancer cell sensitivity to tumor-reactive T cells

**Figure 2:**
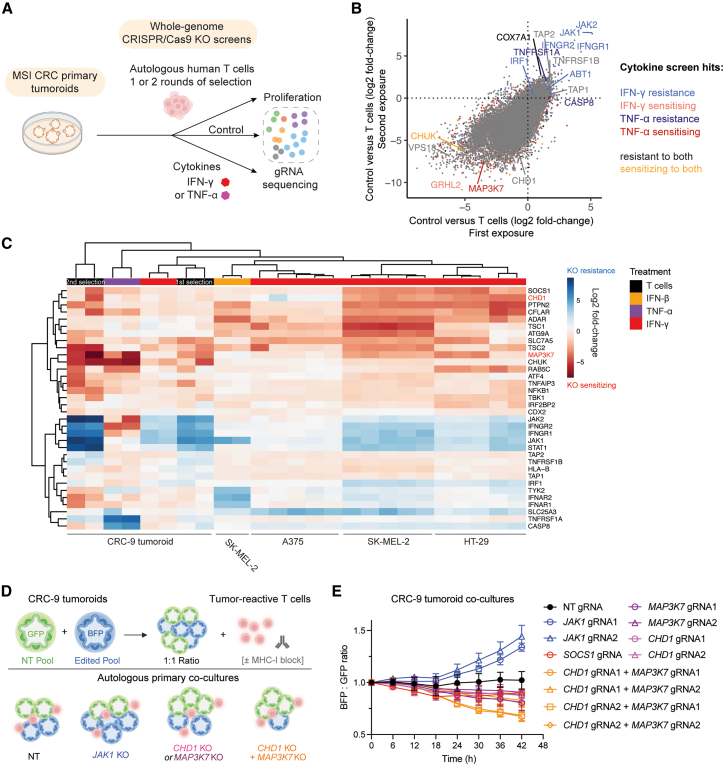
Autologous tumoroid-T cell co-culture CRISPR screens identify modulators of sensitivity to tumor-reactive T cells (A) Overview of the autologous co-culture CRISPR screens with primary tumoroids (CRC-9) and anti-tumor T cells. Tumoroid screens were performed ± IFN-γ (200 ng/mL) or TNF-*α* (100 ng/mL), or in the presence of tumor-reactive T cells (1:1 effector:target ratio) for 10 days. Tumoroids underwent one or two rounds of selection with T cells. MSI CRC, microsatellite-unstable colorectal cancer. (B) Genetic modulators of cancer cell sensitivity to autologous human tumor-reactive T cells. Scatterplot comparing CRISPR KO screen log2-fold change (control versus T cells) from the first and second rounds of T cell selection. Data are representative of two independent screens performed on separate days. *CHD1* and selected co-culture hits from cytokine tumoroid screens are highlighted (*P* adjusted < 0.05). Pearson correlation, *r* = 0.68. (C) Heatmap displaying clustering of cell models and immunological selection pressures based on CRISPR KO screen log2 fold-changes. Columns represent different CRISPR screens against the control sample (e.g., control versus interferon or WT versus *JAK1* KO in the presence of interferon). Genes include published resistance and sensitizing hits and all reach a significance threshold of *P* < 0.05 in at least one experimental condition shown. See also Figure S4B. (D) Overview of a competition assay using patient-derived CRC tumoroids co-cultured with autologous tumor-reactive T cells. The ratio of CRISPR-Cas9-edited (BFP^+^ and mCherry^+^) and non-targeting gRNA-harboring (GFP^+^ and mCherry^+^) tumoroids was monitored over time. (E) *CHD1* and *MAP3K7* loss additively sensitize cancer cells to killing by autologous T cells. Fluorescence of the different cell populations in the competition assay was measured using an Incucyte. Data represent the mean ± SD of two independent experiments, each performed in technical triplicate. Two-way analysis of variance (ANOVA); ∗∗∗∗*P* < 0.0001 (NT versus *CHD1* gRNA1 + *MAP3K7* gRNA1, NT versus *CHD1* gRNA2 + *MAP3K7* gRNA2, NT versus *CHD1* gRNA1 + *MAP3K7* gRNA2, NT versus *CHD1* gRNA2 + *MAP3K7* gRNA1, *CHD1* gRNA1 versus *CHD1* gRNA1 + *MAP3K7* gRNA1, *MAP3K7* gRNA1 versus *CHD1* gRNA1 + *MAP3K7* gRNA1, *CHD1* gRNA2 versus *CHD1* gRNA2 + *MAP3K7* gRNA1, *MAP3K7* gRNA1 versus *CHD1* gRNA2 + *MAP3K7* gRNA1; ∗∗*P* = 0.0012, *MAP3K7* gRNA2 versus *CHD1* gRNA2 + *MAP3K7* gRNA2; ∗*P* = 0.04, *MAP3K7* gRNA2 versus *CHD1* gRNA1 + *MAP3K7* gRNA2; n.s. (not significant) *CHD1* gRNA2 versus *CHD1* gRNA2 + *MAP3K7* gRNA2, *CHD1* gRNA1 versus *CHD1* gRNA1 + *MAP3K7* gRNA2.

Stimulation with individual cytokines facilitates the investigation of specific signaling pathways involved in anti-tumor immunity but cannot recapitulate the combination of factors present at the synapse between T cells and tumor cells. To investigate the genetic determinants of tumor cell sensitivity to tumor-reactive T cells more directly, we established a co-culture system for CRISPR-Cas9 KO screening comprising primary tumoroids derived from an MSI CRC (CRC-9) and autologous tumor-reactive T cells (predominantly CD8^+^ T cells^72^) from peripheral blood mononuclear cells (STAR Methods^12^^,^^73^^,^^74^^,^^75^). To achieve genome-scale screening with primary material, we used a condensed and efficiency-optimized gRNA library, MinLibCas9.^36^ In addition, we screened CRC-9 tumoroids in the presence of IFN-γ and TNF-*α*, cytokines involved in T cell-mediated killing, to deconvolute the contributions of these factors in cancer cell death^76^ (Figure 2A).

Integrating tumoroid-T cell co-culture cytokine screens (Figure S4A) revealed that sensitivity to tumor-reactive T cells was highly dependent on IFN-γ signaling (*JAK1, JAK2, IFNGR1, IFNGR2, STAT1*), with the exception of the TNF-*α* receptor (*TNFRSF1A*, *TNFRSF1B*; Figure 2B). We also identified cytokine-independent hits in T cell screens, such as *TAP1/2* KO, which was associated with resistance; these genes are essential for antigen processing and presentation^77^ (Figures 2B, 2C, S4B, and S4C). In addition, KO of a proposed endogenous neoantigen in this model, mutant *ABT1* (but not *EEF1A1*^72^), conferred resistance to T cells in one of two T cell batches. Independent tumoroid screens with different batches of T cell cultures resulted in lower levels of screen replicate correlation (*r* = 0.31–0.32) than cytokine selection screens (*r* = 0.63–0.53), likely due to different levels of tumor cell killing between T cell batches (Figures S4A and S4B). However, two rounds of tumoroid selection with the same T cell preparations generated more consistent data (*r* = 0.68–0.63), with increasing effect sizes from the first to the second round of cytotoxic selection with T cells (Figure S4B).

Notably, *CHD1* KO was not significantly depleted with T cell treatment, but *MAP3K7* KO sensitized CRC-9 tumoroids to anti-tumor T cells to a similar or greater extent than known regulators of anti-tumor immunity, such as *SOCS1*,^12^ *ADAR*,^78^ *PTPN2*,^10^ and *TBK1*^79^ (Figures 2B and 2C). *MAP3K7* was in the top 1% of sensitizing hits in TNF-*α* screens, implying TNF-*α* dependency in this model. *CHUK* encodes IKK*α*, part of the IKK complex with MAP3K7 (TAK1) that regulates NF-*κ*B activity.^34^ *CHUK* KO also sensitized cells to T cells, TNF-*α*, and IFN-γ, emphasizing the importance of NF-*κ*B signaling in tumor cell susceptibility to T cells (Figure 2C). In co-culture cell competition assays (Figure 2D), both *CHD1* and *MAP3K7* KO sensitized cells to T cell-mediated killing to a similar degree as *SOCS1* KO (Figure 2E). Sensitization was enhanced in dKO tumoroids, perhaps explaining why *CHD1* KO alone was not a significant hit in genome-wide T cell co-culture screens. Furthermore, dKO tumoroids were more sensitive to IFN-γ than WT controls (Figure S4D). Collectively, these data suggest that *CHD1* and *MAP3K7* loss additively enhances cancer cell sensitivity to tumor-reactive T cells.

### *CHD1* and *MAP3K7* control the transcriptional response to IFN-γ

**Figure 3:**
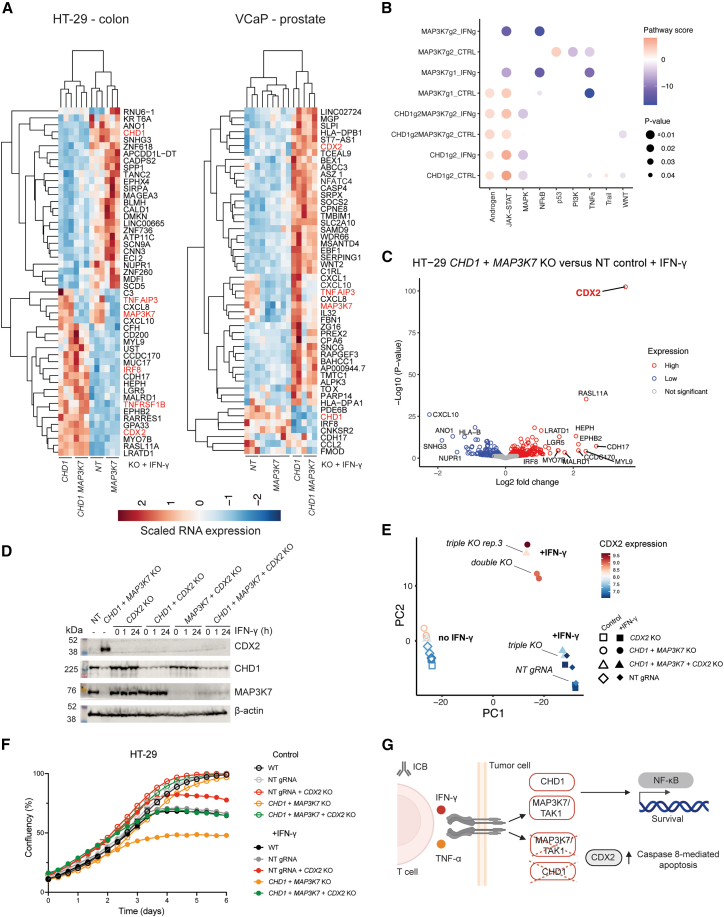
CHD1 and MAP3K7 control the response to IFN-γ in cancer cells through a transcriptional network dependent on CDX2 (A) RNA sequencing analysis reveals transcriptional programs dependent on CHD1 and MAP3K7 in response to IFN-γ. Heatmap and hierarchical clustering of gene expression changes in *CHD1*, *MAP3K7*, and dKO HT-29 and VCaP cells. Relative gene expression values are scaled normalized read counts from DESeq2. Columns represent independent biological replicates, with three biological replicates for each genotype, except for VCaP *CHD1* KO (two replicates; STAR Methods) and VCaP *MAP3K7* KO (six replicates; three replicates for two gRNAs). Differentially expressed transcripts of interest are highlighted in red for the top 50 significant transcripts (*P*-adjusted value < 0.05) and *CDH17*, *IRF8*, *CDX2*, and *MAP3K7* for comparison across cancer cell models. (B) Pathway analysis reveals altered NF-*κ*B, JAK-STAT, and androgen receptor signaling in *CHD1* and *MAP3K7* KO VCap cells. Pathway activity for single and dKO cells ± IFN-γ (500 U/mL) for 24 h. *p* values and pathway scores are derived from decoupleR-PROGENy. (C) *CDX2* expression is upregulated in dKO HT-29 cells. Volcano plot of differentially expressed genes comparing dKO and NT gRNA-harboring HT-29 cells cultured in IFN-γ (500 U/mL) for 24 h. Data represent the average of three independent biological replicates. (D) Confirmation of *CHD1* and *MAP3K7* KO and upregulation of *CDX2* in dKO cells. Western blotting of HT-29 cells with the indicated KO ± IFN-γ (500 U/mL) for 1 or 24 h. Results are representative of two independent experiments. (E) *CDX2* KO reverses IFN-γ-induced transcriptional programs in dKO cells. Principal component analysis comparing normalized RNA counts from *CDX2* KO, *CHD1*, and *MAP3K7* dKO, triple KO (*CDX2* + *CHD1* + *MAP3K7* KO), and NT gRNA-harboring HT-29 cells grown ± IFN-γ (500 U/mL) for 72 h before analysis. 91% and 7% of the variance are explained by PC1 and PC2, respectively. (F) KO of *CDX2* rescues the IFN-γ sensitization effect of *CHD1* and *MAP3K7* KO in HT-29 cells. Cell proliferation was monitored ± IFN-γ (500 U/mL) using an Incucyte. Data represent the mean ± SD of three technical replicates and are representative of two independent experiments. (G) Model outlining how tumor cell *CHD1* and *MAP3K7* loss coordinately sensitizes cells to T cells and lymphocyte-derived cytokines. *CHD1* and *MAP3K7* loss alters the cancer cell transcriptional response to cytokines through upregulation of the transcription factor CDX2 and reduced NF-*κ*B signaling.

To investigate the mechanism through which *CHD1* and *MAP3K7* loss sensitizes cells to IFN-γ and anti-tumor T cells, we generated single and dKO HT-29 and VCaP cancer cell models (Figures S3C and S3F) and performed RNA sequencing (RNA-seq) in the presence and absence of IFN-γ (Figure S5A). This analysis confirmed decreased expression of *CHD1* and *MAP3K7* in KO samples, implying nonsense-mediated decay (Figure 3A), and revealed the broad transcriptional impact of *CHD1* KO, reflecting its role in chromatin remodeling.^30^ In HT-29, *CHD1* KO increased the expression of *IRF8*, a master regulator of IFN-γ signaling,^80^ and *TNFRSF1B*, which encodes part of the TNF-*α* receptor. *MAP3K7* KO reduced *TNFAIP3* and *CXCL10* expression in both cell models, suggesting a degree of mechanistic overlap between tissue types and consistent with a conserved role for *MAP3K7* in NF-*κ*B signaling.^34^ Pathway analysis^81^^,^^82^ revealed enrichment in genes related to JAK-STAT signaling in single KO and dKO VCaP cells (Figure 3B), although this was not the case in HT-29 cells, which displayed varying levels of decreased JAK-STAT signaling (Figure S5B). TNF-*α* signaling via NF-*κ*B was the most downregulated pathway from gene set enrichment analysis (GSEA) of HT-29 dKO cells treated with IFN-γ for 72 h, but this pathway was significantly upregulated in VCaP cells, reflecting tissue-specific effects (Table S3). However, both cell models displayed a decrease in NF-*κ*B signaling mediated through *MAP3K7* KO (Figures 3B and S5B). Androgen receptor signaling was significantly upregulated in *MAP3K7* KO and *CHD1* KO conditions in VCaP (Figure 3B), implying a convergent mechanism and consistent with the frequent co-deletion of these genes in prostate cancer^21^^,^^24^ (Figure 3B). Transcriptional signatures of increased androgen receptor signaling following loss of either or both genes (Figure 3B) could explain why they are most frequently mutated in prostate cancer and associated with resistance to anti-androgen treatment.^23^ Recent studies further support the unanticipated link between cancer cell JAK-STAT and androgen receptor signaling.^83^^,^^84^

### CDX2 mediates IFN-γ sensitivity induced by *CHD1* KO

The most significant gene expression change in IFN-γ-stimulated dKO HT-29 cells compared to non-targeting (NT) gRNA control cells was the induction of *CDX2* (Figure 3C), a transcription factor involved in NF-*κ*B signaling, tissue inflammation, and development.^85^ Notably, CDX2-positive CRC has a better prognosis^86^ and is associated with higher ICB response rates,^87^ and *CDX2* KO caused resistance to IFN-γ in CRC-9 tumoroid screens (Figure S4B). Increased *CDX2* expression was predominantly driven by *CHD1* KO, with some contribution from *MAP3K7* KO (Figure S3D), and was evident in both cell models but more pronounced in HT-29. This could reflect tissue-specific differences in baseline *CDX2* expression, which were higher in VCaP cells (Figures S3D, S5C, and S5D). Western blot analysis verified the induction of CDX2 in *CHD1* and *MAP3K7* KO HT-29 and VCaP cells at the protein level (Figures 3D, S3D, and S5D).

To investigate the potential role of CDX2 in regulating the IFN-γ response, we generated a triple KO (tKO) HT-29 cell model deficient in *CDX2* (Figure 3D). RNA-seq analysis of *CDX2* KO cells revealed significant downregulation of *IRF8* (Figure S5E) and the IFN-γ pathway in GSEA (Figure S5F). Principal component analysis of RNA expression revealed clustering by genotype, with tKO cells clustering with NT gRNA control samples, implying partial reversion of the induced transcriptional signature in dKO cells (Figure 3E). One tKO biological replicate (rep.3) had higher levels of *CDX2* (Figure S5G) and clustered with dKO samples (Figure 3E), suggesting that a threshold level of *CDX2* expression is required to sustain this transcriptional program.

Transcription factor analysis highlighted increased CDX2 activity in dKO cells, reduced NF-*κ*B activity (REL, RELA, NFKB1), and decreased activity of transcription factors involved in vesicular trafficking, autophagy (ATF2, ATF4), and IRF/STAT (Figure S6A). Consistently, GSEA revealed increased mTORC pathway activity and decreased autophagy—a known immune evasion mechanism—^13^ which was reversed with *CDX2* KO (Figure S6B). Moreover, *CDX2* KO reversed gene programs induced in the dKO genotype, including TNF-*α* signaling via NF-*κ*B (Figures S6C and S6D). *CDX2* KO was protective against IFN-γ in VCaP cells but could not fully reverse IFN-γ sensitization in the dKO context (Figure S6E). In contrast, *CDX2* KO fully rescued sensitization to IFN-γ in HT-29 (Figure 3F). tKO cells had comparable sensitivity to IFN-γ as NT gRNA control cells, consistent with *CDX2* playing a key role in sensitizing HT-29 cells to IFN-γ (Figure 3G).

### *Chd1* and *Map3k7* deletion enhances anti-tumor immunity and response to ICB in a mouse model

**Figure 4:**
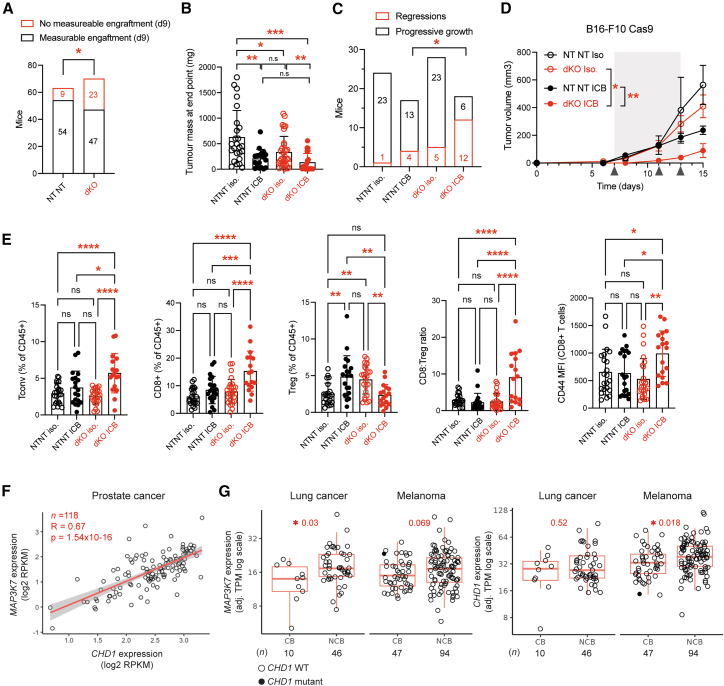
Reduced *CHD1* and *MAP3K7* expression enhances anti-tumor immunity and correlates with clinical response to immune checkpoint blockade (A) *Chd1* and *Map3k7* dKO B16-F10 tumor cells have a higher frequency of spontaneous rejection. Engraftment rates of NT gRNA-harboring or dKO B16-F10 cells injected subcutaneously into syngeneic C57BL/6 mice at day 9 post-injection. Data represent four independent experiments. Two-sided Fisher’s exact test; ∗*P* = 0.015. (B) *Chd1* and *Map3k7* dKO B16-F10 tumors grow more slowly *in vivo*. Endpoint tumor mass of successfully engrafted B16-F10 NT gRNA and dKO tumors in mice treated with intraperitoneal anti-PD-1 and anti-CTLA-4 immune checkpoint blockade (ICB) or isotype (Iso.) control. Data represent the mean ± SD from three independent experiments. Unpaired, two-tailed Student’s *t* test; *n* = 25 NT NT iso.; *n* = 18 NT NT ICB; *n* = 33 dKO iso.; *n* = 21 dKO ICB. ∗∗∗*P* = 0.0002, ∗∗*P* = 0.0025 NT NT iso. versus NT NT ICB, ∗∗*P* = 0.0094 dKO iso versus dKO ICB, ∗*P* = 0.01. (C) Tumor regressions are more frequent in *Chd1* and *Map3k7* dKO B16-F10 tumors treated with ICB. Subcutaneous tumors were measured using calipers, and tumors with a decrease in volume were considered regressions. Data are pooled from three independent experiments. NT NT ICB versus dKO ICB, two-sided Fisher’s exact test; ∗*P* = 0.0176. (D) Improved immunotherapy response in *Chd1* and *Map3k7* dKO B16-F10 tumors. Growth curves of subcutaneously engrafted B16-F10 NT NT gRNA or dKO tumors ± ICB. Data represent the mean ± SEM and are representative of three independent experiments. Two-way analysis of variance (ANOVA); ∗*P* = 0.0094; ∗∗*P* = 0.0019. dKO Iso *n* = 6, dKO ICB *n* = 5, NT NT Iso *n* = 4, NT NT ICB *n* = 6. (E) Heightened anti-tumor immunity in *Chd1* and *Map3k7* dKO B16-F10 tumors treated with ICB. Immunoprofiling of subcutaneous tumors at the endpoint (days 15–17). Relative abundance of intratumoral conventional CD4^+^ T cells, CD8^+^ T cells, regulatory T cells (T_regs_), and the CD8^+^ T cells:T_reg_ ratio, as well as the surface expression of the activation marker CD44 on CD8^+^ T cells, was assessed by flow cytometry. MFI, mean fluorescence intensity. Data represent the mean ± SD and are pooled from three independent experiments. One-way ANOVA; ∗∗∗∗*P* < 0.0001; ∗∗∗*P* < 0.0005; ∗∗*P* < 0.01; ∗*P* < 0.05; ns = not significant. NT NT Iso. *n* = 22 or *n* = 23 for CD8 analysis. NT NT ICB *n* = 20 or *n* = 19 for Tconv. analysis. dKO Iso. *n* = 25 or *n* = 27 for CD8 analysis. dKO ICB *n* = 17 or *n* = 16 for CD8:Treg or *n* = 18 for CD44 analysis. (F) Correlation of *CHD1* and *MAP3K7* mRNA expression in prostate cancers^92^ expressed as log2 fragments per kilobase of transcript per million (FMPK). Spearman’s rank correlation, R = 0.67, *P* = 1.54e−16. (G) Reduced expression of *CHD1* and *MAP3K7* in lung cancer and melanoma is associated with clinical response to ICB. Boxplot displaying tumor *CHD1* and *MAP3K7* mRNA expression (adjusted transcripts per million; adj. TPM) and clinical responses to ICB in patients from the Hartwig Medical Foundation.^93^ Significance was assessed using the Wilcoxon signed-rank test, and *n* denotes the number of patients. Boxplots represent the median and interquartile range (IQR), and whiskers indicate the lowest and highest values within 1.5 × IQR. CB, clinical benefit; NCB, no clinical benefit.

To assess the potential relevance of our findings *in vivo*, we selected a mouse model of melanoma, B16-F10, due to its immunogenicity and well-validated response to immunotherapy.^88^ We generated a B16-F10 model deficient in *Chd1* and *Map3k7* (Figure S7A) and subcutaneously engrafted control cells (expressing an NT gRNA) or dKO cells into syngeneic C57BL/6 mice. dKO cells grew at a comparable rate to control cells *in vitro* (Figure S7B), in line with reports demonstrating that single KO and dKO of *CHD1* and *MAP3K7* do not have an altered proliferation phenotype in immunodeficient mice.^32^^,^^89^^,^^90^^,^^91^ However, engraftment rates were lower in immunocompetent mice (Figure 4A), and dKO cells grew slower *in vivo* compared to controls (Figure 4B), consistent with a non-cell-autonomous anti-tumor effect.

To test whether loss of tumor cell *Chd1* and *Map3k7* would alter responses to ICB, we treated tumor-bearing mice with a combination of anti-PD-1 and anti-CTLA-4 monoclonal antibodies to mimic ICB therapy regimes in patients with melanoma.^38^ We verified that systemic ICB was functional by measuring dendritic cell influx into inguinal, tumor-draining lymph nodes using flow cytometry (Figures S7C–S7F). Both dKO and control tumors responded to ICB; however, dKO tumors regressed more frequently (Figure 4C) and grew slower than control tumors under treatment (Figure 4D). This was associated with an increase in intratumoral CD8^+^ T cells and conventional CD4^+^ T cells and a reduction in CD4^+^Foxp3^+^ regulatory T cells (T_regs_) in dKO tumors compared with control tumors treated with ICB, resulting in an elevated CD8^+^:T_reg_ ratio (Figure 4E). Moreover, ICB-treated dKO tumors displayed an increase in activated (CD44^+^) intratumoral CD8^+^ T cells. Despite previous reports suggesting that *Chd1* loss in tumors can affect myeloid-derived suppressor cell (MDSC) recruitment,^32^ we did not observe a change in MDSC frequencies (Figure S8F), possibly due to the low overall abundance of MDSCs in these B16-F10 tumors. Overall, these data indicate a heightened anti-tumor adaptive immune response following ICB (Figure 4E).

### Reduced *CHD1* and *MAP3K7* expression correlates with response to ICB in patients

To evaluate the potential clinical relevance of our findings, we compared tumor *CHD1* and *MAP3K7* mRNA expression levels in patient samples. In prostate tumors, where dysregulation of these genes is most prevalent,^22^ *CHD1* and *MAP3K7* mRNA expression were highly correlated (R = 0.67), reinforcing the concept of co-regulation^92^ (Figure 4F). Interestingly, *JAK2* was significantly co-expressed with *CHD1* and *MAP3K7* (Figure S8A), implying coregulation with key IFN-γ pathway genes. In patient records from the Hartwig Medical Foundation^93^ (HMF), *CHD1* and *MAP3K7* mRNA expression in tumors was significantly correlated with clinical responses to ICB, including anti-PD1/PD-L1 and anti-CTLA-4 therapies (Figure 4G, Methods). Individuals with lung cancer who derived clinical benefit from ICB had lower tumor *MAP3K7* expression (*P* = 0.03), and individuals with melanoma who derived clinical benefit from ICB had lower tumor *CHD1* expression than non-responders (*P* = 0.018), outperforming known biomarkers of response such as *CD274* (PD-L1) and *CD8A* in lung cancer and *CCND1*^94^ in lung cancer and melanoma (Figures S8B–S8E), although this trend was not significant in urothelial tumors (Figure S8C). Notably, *CDX2* tumor expression was not significantly correlated with ICB outcome (*p* > 0.14, Wilcoxon signed-rank test). *CHD1* and *MAP3K7* expression were modestly correlated in tumor samples from patients treated with ICB (*R* = 0.47–0.19; Figure S8D), and expression was not correlated with tumor purity, implying this association is independent of tumor immune cell content (Figure S8E). Taken together, these data suggest that reduced tumor *CHD1* and *MAP3K7* expression could serve as biomarkers of ICB response.

## Discussion

Here, we present a functional genomics landscape of genetic dependencies in four cancer cell models in the context of diverse immunological selection pressures, comprising 155 CRISPR screening samples. To map genetic determinants of sensitivity to T cells, we developed a co-culture CRISPR screening platform using autologous, tumor-reactive T cells and primary tumoroids expressing endogenous tumor neoantigens. Our co-culture screening platform is more scalable than *in vivo* screens^95^ and, by using primary human material, accounts for potential cross-species differences.^96^

Integrated analysis of CRISPR-Cas9 KO screens across cancer cell models and different cytokines facilitated deconvolution of the contributions of each cytokine to tumor cell killing and identified shared and private genetic modulators of response. We identify a conserved role for amino acid sensing and mTOR signaling in mediating cancer cell sensitivity to cytokines. mTOR inhibits autophagy, providing a functional link to a known immune evasion pathway,^13^ involved in the lysosomal destruction of pro-death complexes formed in response to cytotoxic cytokines.^97^ These results imply that rapamycin could exert part of its immunosuppressive effect by acting directly on tissues rather than on the immune cell compartment alone.^98^

We identified an acquired vulnerability in tumor cells that have lost *CHD1* and *MAP3K7* expression—enhanced sensitivity to IFN-γ, TNF-*α*, and tumor-reactive T cells. These findings are complementary to a recent report identifying *MAP3K7* as a cancer cell checkpoint against T cell-mediated killing.^91^ We find that *CHD1* and *MAP3K7* control cancer cell transcriptional responses to IFN-γ, at least in part, by regulating the expression of the transcription factor CDX2, and RNA-seq data indicate that differing levels of *CDX2* expression might result in tissue-specific differences. *CHD1* and *MAP3K7* deletion has been shown to impact interferon response gene expression and sensitivity to oncolytic viruses in prostate cancer cells.^89^ MAP3K7 and CHD1 are involved in activating NF-*κ*B signaling^99^ and transcription,^32^ and CDX2 is itself regulated by NF-*κ*B.^85^ Collectively, these findings support a mechanism whereby *CHD1* and *MAP3K7* loss reduces NF-*κ*B signaling in response to cytokines, thereby priming cancer cells for apoptosis^100^^,^^101^ (Figure 3G). *MAP3K7* loss is also associated with RIPK1-mediated apoptosis in the presence of TNF-α.^102^ CHD1 and MAP3K7 inhibition could be attractive options for future combination therapies with ICB. Although selective inhibitors for CHD1 have only recently been described,^103^ several MAP3K7 (TAK1) inhibitors exist, such as takinib^71^^,^^104^ and 5(Z)-7-oxozeaenol,^105^ and have been developed to treat inflammatory diseases, including rheumatoid arthritis. Notably, the TAK1 inhibitor HS-276 has recently been shown to selectively kill glioma stem cells with elevated IFN signaling.^106^ However, potential off-target effects of these inhibitors and the profound impact of MAP3K7 deletion on T and B cell signaling^107^^,^^108^ warrant caution when considering systemic administration with ICB.

In summary, the functional genomics dataset presented here provides a rich resource for investigating the networks underlying cytokine signaling in inflammatory diseases and cancer immunity and highlights *CHD1* and *MAP3K7* loss as a potential biomarker of ICB response, thereby presenting future opportunities to improve cancer immunotherapy outcomes.

### Limitations of the study

The *in vitro* autologous co-culture screens presented here capture the biology of human T cell interactions with tumor cells but cannot recapitulate other complex features and cell-cell interactions in the cancer immunity cycle *in vivo.*^109^ The limited availability of suitable syngeneic, highly immunogenic prostate tumor models restricted our exploration of potential prostate-specific effects of *CHD1* and *MAP3K7* loss on tumor immunity and ICB response *in vivo*. Lastly, the assessment of *CHD1* and *MAP3K7* mutation or expression status as biomarkers of immunotherapy response could be further explored in prospective trials across different cancer types with high frequencies of *CHD1* and *MAP3K7* alterations. Determining the specific effects of *MAP3K7* loss in these studies will be important, as *MAP3K7* is often lost in the context of larger deletions on chromosome 6q.^29^

## Resource availability

### Lead contact

Requests for further information and reagents should be directed to and will be fulfilled by the lead contact, Matthew A. Coelho (matthew.coelho@sanger.ac.uk).

### Materials availability

Reagents generated in this study are available from the lead contact upon request.

### Data and code availability

• DNA and RNA sequencing data are deposited on ENA, and accessions are listed in Table S6, including ERP145139, ERP145138, ERP168780, ERP142756, ERP168941, ERP140862, ERP141157, ERP148434, ERP146719, and ERP171215. All raw CRISPR read counts can be found in Table S7.
• All collective code used to analyze CRISPR-Cas9 KO screens can be found here: https://doi.org/10.5281/zenodo.17856269 and here https://github.com/MatthewACoelho/. Individual code is also available on GitHub. For example, to analyze RNA-seq data for differential expression and principal component analysis: https://github.com/ABWatterson/DESeq2_All_RNAseq_PCA. To analyze RNA-seq data for PROGENy model-based pathway activation (CollecTRI network): https://github.com/ABWatterson/decoupleR-Pathway-activation-main. To analyze RNA-seq data for CollecTRI network-based transcription factor activation: https://github.com/ABWatterson/decoupleR-TF-activation.
• Any additional information required to reanalyze the data reported in this paper is available from the lead contact upon request.

## Acknowledgments

This research was funded in whole or in part by the 10.13039/100010269Wellcome Trust (grant no. 206194) and by Open Targets (OTAR2061). The authors acknowledge the contribution of the Cancer Aging and Somatic Mutation Support team at the Wellcome Sanger Institute. Figure components were created with BioRender.com. This work was supported by 10.13039/501100000289Cancer Research UK [RCCCDF-Nov23/100002] and partly funded by a Sanger Accelerator Award for Postdocs, which included a contribution from Sanger’s portion of the UKRI Talent & Research Stabilisation Fund. For the purpose of Open Access, the author has applied a CC BY public copyright license to any author-accepted manuscript version arising from this submission. We thank Olli Dufva and Saroor Patel for critical reading of the manuscript and the Cancer Genome Editing laboratory for feedback on the manuscript. This publication and the underlying study were made possible in part by data provided by the Hartwig Medical Foundation and the Center of Personalised Cancer Treatment (CPCT) through the Hartwig Medical Database. Data for this study were also provided in part by the Netherlands Cancer Institute (Antoni van Leeuwenhoek Ziekenhuis). Additionally, patient results published here are based in part upon data generated by the Therapeutically Applicable Research to Generate Effective Treatments (TARGET) initiative, phs000218.v24.p8, managed by the NCI (available at National Cancer Institute (NCI) TARGET: Therapeutically Applicable Research to Generate Effective Treatments), the TCGA Research Network (https://www.cancer.gov/tcga), and the The Metastatic Breast Cancer Project and The Metastatic Prostate Cancer Project projects of Count Me In. The authors thank all patients, medical staff, and research staff for making this work possible.

## Author contributions

M.A.C., M.J.G., T.Y.F.H., and E.E.V. devised the study. A.W. performed CRISPR and RNA-seq experiments and assisted with RNA-seq analysis. G.P. and S.C. devised tumoroid experiments, and G.P., S.F.V., and A.W. executed tumoroid experiments. E.K., S.B., A.W., and M.A.C. analyzed CRISPR screening and RNA-seq data. V.V. and C.M.C. developed tumoroid and autologous T cell reagents and protocols. T.W.B. assisted with patient data analysis. Y.S. assisted with *in vivo* experiments, flow cytometry, and analysis, and T.Y.F.H. advised on *in vivo* experiments and analysis. M.A.C. performed HMF and CRISPR analysis. Funding acquisition was by M.A.C., M.J.G., and E.E.V. M.A.C. and M.J.G. wrote the manuscript, and all authors reviewed the manuscript.

## Declaration of interests

M.A.C. and M.J.G. are cofounders of BASE Rx. M.J.G. has received research grants from AstraZeneca, GlaxoSmithKline, and Astex Pharmaceuticals and is a founder and advisor for Mosaic Therapeutics. E.E.V. is a founder and advisor for Mosaic Therapeutics.

## STAR★Methods

### Key resources table

**Table:** 

REAGENT or RESOURCE	SOURCE	IDENTIFIER
**Antibodies**		

Anti-human PD-1	BioXCell	Cat.# BP0146; RRID: AB_2894808
Anti- human CTLA4	BioXCell	Cat.# BE0032
Anti-human PD-1 Isotype control	BioXCell	Cat.# BP0089; RRID: AB_2894744
Anti-human CTLA4 Isotype control	BioXCell	Cat.# BE02060
Anti-mouse IgG horseradish peroxidase	Amersham	Cat.# NXA931V
Anti-rabbit IgG horseradish peroxidase	Amersham	Cat.# NA934V
Anti-mouse CD16/32 (TruStain FcX)	Biolegend	Cat.# 101320; RRID: AB_1574975
Anti-mouse CD45-BV510	Biolegend	Cat.# 103137; RRID: AB_2561392
Anti-mouse CD3e-PeCy7	Thermo Fisher	Cat.# 25-0031-82; RRID: AB_469572
Anti-mouse B220-APC-eFluor 780	Thermo Fisher	Cat.# 47-0452-82; RRID: AB_1518810
Anti-mouse CD4-AF700	eBioscience	Cat.# 56-0041-82; RRID: AB_493999
Anti-mouse CD8a-BB700	BD Biosciences	Cat.# 566409; RRID: AB_2744467
Anti-mouse NK1.1-BUV395	BD Biosciences	Cat.# 564144; RRID: AB_2738618
Anti-mouse FceRIa-eFluor450	eBioscience	Cat.# 48-5898-82; RRID: AB_2574086
Anti-mouse CD11b-eFluor450	eBioscience	Cat.# 48-0112-82; RRID: AB_1582236
Anti-mouse CD11c-eFluor450	eBioscience	Cat.# 48-0114-82; RRID: AB_1548654)
Anti-mouse F4/80-eFluor450	eBioscience	Cat.# 48-4801-82; RRID: AB_1548747
Anti-mouse Ly6G-eFluor450	eBioscience	Cat.# 48-9668-82; RRID: AB_2637124
Anti-mouse Ly6C-eFluor450	eBioscience	Cat.# 48-5932-82; RRID: AB_10805519
Anti-mouse CD3-eFluor450	eBioscience	Cat.# 48-0031-82; RRID: AB_10735092
Anti-mouse NK1.1-eFluor450	eBioscience	Cat.# 48-5941-82; RRID: AB_2043877
Anti-mouse CD5-eFluor450	eBioscience	Cat.# 48-0051-82; RRID: AB_1603250
Anti-mouse CD19-eFluor450	eBioscience	Cat.# 48-0193-82; RRID: AB_2734905
Anti-mouse B220-eFluor450	eBioscience	Cat.# 48-0452-82; RRID: AB_1548761
Anti-mouse FceRIa-PerCP-eFluor710	eBioscience	Cat.# 46-5898-82; RRID: AB_2573801
Anti-mouse CD172α-AF488	Biolegend	Cat.# 144024; RRID: AB_2650814
Anti-mouse Siglec-F SB600	eBioscience	Cat.# 63-1702-82; RRID: AB_2688074
Anti-mouse XCR1 BV650	Biolegend	Cat.# 148220; RRID: AB_2566410
Anti-mouse CD64-BV711	Biolegned	Cat.# 139311; RRID: AB_2563846
Anti-mouse CD11b-BV785	Biolegend	Cat.# 101243; RRID: AB_2561373
Anti-mouse I-A/I-E-BUV395	BD Biosciences	Cat.# 569244; RRID: AB_3684900
Anti-mouse CD11c-AF700	eBioscience	Cat.# 56-0114-82; RRID: AB_493992
Anti-mouse F4/80-APC-eFluor780	eBioscience	Cat.# 47-4801-82; RRID: AB_2735036
Anti-mouse Ly6G-PE-eFluor610	eBioscience	Cat.# 61-9668-82; RRID: AB_2574679
Anti-mouse Ly-6C-PE-Cy7	eBioscience	Cat.# 25-5932-82; RRID: AB_2573503
β-tubulin	Sigma Aldrich	Cat.#T4026; RRID: AB_477577
β-actin	CellSignalling	Cat.# 4970; RRID: AB_2223172
CHD1	CellSignalling	Cat.# 4351; RRID: AB_11179073
MAP3K7	CellSignalling	Cat.# 45206
STAT1	CellSignalling	Cat.# 9172; RRID: AB_2198300
pSTAT1-Y710	CellSignalling	Cat.# 7649; RRID: AB_10950970
JAK1	CellSignalling	Cat.# 3344; RRID: AB_2265054
JAK2	CellSignalling	Cat.# 3230; RRID: AB_2128522
Cas9	CellSignalling	Cat.# 14697; RRID: AB_2750916
CDX2	CellSignalling	Cat.# 3977; RRID: AB_2077043
Vinculin	CellSignalling	Cat.# 13901; RRID: AB_2728768
Cleaved caspase 3	CellSignalling	Cat.# 9661; RRID: AB_2341188
Caspase 8	CellSignalling	Cat.# 9746; RRID: AB_2275120

**Bacterial and virus strains**		

Lucigen Endura™ ElectroCompetent Cells	BioSearch Technologies	Cat.# 60242-1
Stable Competent *E*. *coli*	NEB	Cat.#C3040I

**Biological samples**		

CRC-9 tumor organoid and autologous PBMC cultures	Cattaneo et al.^74^	N/A

**Chemicals, peptides, and recombinant proteins**		

IFN-γ	Thermo Fisher	Cat.# 300-02-100 μg
IFN-β	STEMCELL Technologies	Cat.# 78113.1
TNF-α	Thermo Fisher	Cat.# PHC3011
IL-6	Thermo Fisher	Cat.# PHC0064
Basement membrane extract (BME)	R&D Systems	Cat.# 3433-010-R1
Fixable viability dye - UV 455	eBioscience	Cat.#

**Critical commercial assays**		

CellTitre-Glo	Promega	Cat.#G7570
Foxp3/Transcription Factor Kit	Thermo Fisher	Cat.# 00-5521-00

**Deposited data**		

CRISPR-Cas9 screens SK-MEL-2	This paper	ENA: ERP145139
CRISPR-Cas9 screens SK-MEL-2	This paper	ENA: ERP145138
CRISPR-Cas9 screens HT-29	This paper	ENA: ERP168780
CRISPR-Cas9 screens HT-29	This paper	ENA: ERP142756
CRISPR-Cas9 screens HT-29	This paper	ENA: ERP168941
CRISPR-Cas9 screens A375	This paper	ENA: ERP140862
CRISPR-Cas9 screens A375	This paper	ENA: ERP141157
RNA sequencing HT-29	This paper	ENA: ERP148434
RNA sequencing VCaP	This paper	ENA: ERP146719
CRISPR-Cas9 screens CRC-9	This paper	ENA: ERP171215

**Experimental models: Cell lines**		

HT-29	NCI	RRID: CVCL_0320
A375	ATCC	RRID: CVCL_0132
SK-MEL-2	NCI	RRID: CVCL_0069
VCaP	ATCC	RRID: CVCL_2235
B16-F10	ATCC	RRID: CVCL_0159

**Experimental models: Organisms/strains**		

Mouse: C57BL/6	Charles River Laboratories	N/A

**Oligonucleotides**		

Primers are listed in Table S4	This paper	N/A
gRNAs are listed in Table S4	This paper	N/A

**Recombinant DNA**		

pSpCas9n(BB)-2A-GFP (PX461)		Addgene #48140
Human MinLibCas9 library		Addgene #164896
psPAX2 lentiviral packaging plasmid		Addgene #12260
pMD2.G lentiviral packaging plasmid		Addgene #12259
pKLV2-EF1a-Cas9Bsd-W		Addgene #68343
pKLV2-EF1a-BsdCas9-W		Addgene #67978
pKLV2-U6gRNA5 (gGFP)-PGKmCherry2AGFP-W		Addgene #67982
pKLV2-U6gRNA5(BbsI)-PGKpuro2ABFP-W		Addgene #67974
pKLV2-EF1a-BsdCas9-W		Addgene #67978

**Software and algorithms**		

TIDE	Brinkman et al.^110^	N/A
Incucyte S3	Sartorius	v2018B
FCSExpress	Dotmatics	N/A
Prism 9	GraphPad	N/A
R	Comprehensive R Archive Network R project	N/A
GSEA	Broad Institute	Version: 4.3.3
Custom analysis code	This paper	https://doi.org/10.5281/zenodo.17856269

### Experimental model and study participant details

#### Animals

*In vivo* experiments were performed under project license number PP7993249 and in line with Cancer Research UK Cambridge Institute institutional guidelines. 10-13 week-old female C57BL/6 mice were used for syngeneic transplantation studies and were randomly assigned to experimental groups. None of the mice were subject to procedures prior to commencing the study. Mice were housed in according to institutional guidelines and in accordance with UK Home Office guidelines. Food and water were supplied *ad libitum* in individually ventilated cages.

#### Primary cell cultures

PBMCs and CRC-9 tumor organoids were from the Netherlands Cancer Institute (NKI). CRC-9 is genetically female. Derivation of tumor organoids, enrichment of tumor-reactive T cell populations from patient PBMCs were performed as described.^74^ Cells were maintained in a 5% CO2, 95% air, humidified incubator at 37°C.

#### Cell lines

All cell lines were mycoplasma tested and verified as mycoplasma-free and STR profiled in accordance with authentication guidelines. Cells were maintained in a 5% CO2, 95% air, humidified incubator at 37°C, in RPMI or DMEM medium with 10% FCS and 1× penicillin-streptomycin or RPMI medium with 10% FCS, 2.5g Glucose, 1× Sodium Pyruvate and 1× penicillin-streptomycin (Thermo Fisher). Human (HT-29, A375, SK-MEL-2, VCaP) and mouse (B16-F10) cancer cell lines used in this study, RRID identifiers, and their source, are listed in the key resources table.

### Method details

This research was conducted in accordance with institutional guidelines at the Wellcome Sanger Institute and Cancer Research UK Cambridge Institute as outlined in the Good Research Practice Guidelines (v4, 2021) and Home Office project license number PP7993249. We support inclusive, diverse, and equitable research.

#### CRISPR-Cas9 cell lines and tumoroids

To generate Cas9-expressing cell lines, cells were transduced overnight with lentivirus containing Cas9 (pKLV2-EF1a-Cas9Bsd-W; Addgene #68343) plus polybrene (8 μg/mL; Thermo Fisher). 24 h post-transduction, lentivirus-containing medium was refreshed with complete medium. 48 h post-transduction, positively transduced cells were selected for with blasticidin (Thermo Fisher). Cas9 activity was determined as described previously.^111^ Briefly, cells were transduced with Cas9 reporter virus (pKLV2-U6gRNA5 (gGFP)-PGKmCherry2AGFP-W; Addgene #67982). The number of BFP^+^ and GFP-mCherry double-positive cells were determined by flow cytometry on a BD LSR Fortessa instrument (BD Biosciences), and data were subsequently analyzed using FCSExpress to determine the percentage of mCherry^+^ cells. For tumoroids, tumoroids were dissociated into single cells and incubated overnight in suspension culture with complete media containing pKLV2-EF1a-BsdCas9-W lentiviral particles and polybrene (8 μg/mL) to express Cas9. The following day, the cells were seeded in BME (R&D systems) and cultured as tumoroids. Blasticidin selection (20 μg/mL) was initiated 48 h post-transduction and continued for the duration of the experiments. The tumoroid demonstrated Cas9 activity exceeding 80%.

#### *JAK1* and *JAK2* KO cell cloning

Cas9 expressing cell lines HT-29, A375 and SK-MEL-2 were plated in a 6-well plate to achieve 80% confluency after 24 h. After 24 h, transient transfection of *JAK1* or *JAK2* gRNAs was achieved by replacing media with 1.9 mL culture media, then combining 100 μL/well of Opti-MEM (Thermo Fisher), 1 μg/well of plasmid (gRNA-GFP, Addgene #48140), and 3 μL/well FuGENE (Promega) which was incubated at room temperature for 15 min then added dropwise into the wells. After 24 h incubation, media was refreshed, and cells incubated for a further 24 h. Cells were then stained with 1 μg/mL DAPI (Sigma-Aldrich) before FACS sorting (DAPI^−^/GFP^+^) sorted as single cells (1 cell per well) into 96-well plates containing their respective advanced media (advanced-RPMI and advanced-DMEM; Thermo Fisher) using a Bigfoot Spectral Cell Sorter (Thermo Fisher). Clones were expanded and assessed for genomic *JAK1* or *JAK2* KO by PCR of the gRNA target site and TIDE analysis.^110^ All primers are listed in Table S4. Successful clones were expanded for 9 days and assessed for GFP expression (Incucyte S3; Sartorius) to ensure a lack of plasmid integration. GFP-negative clones were expanded further and frozen. Protein level KO was confirmed by Western blotting. *JAK1/2* KO, Cas9 protein expression and the abolition of JAK-STAT signaling was assessed by treating clones for 1 h with IFN-γ and IL-6 (400 U/mL and 20ng/mL, respectively) before blotting for JAK1/2, pSTAT1/3, and Cas9. Functional resistance to IFNs was confirmed by Incucyte S3 (Sartorius) proliferation assays over 5–10 days by treating clones with 400 U/mL IFN-γ (Thermo Fisher) or IFN-β (STEMCELL Technologies).

#### Library production

We used the Human MinLibCas9 library (Addgene #164896), which contains 37,722 guides targeting 18,761 protein-coding genes (two guides per gene), with a further 200 non-targeting guides, with no GRCh38 perfect alignment. The vector backbone for the library was modified from pKLV2-U6gRNA(BbsI)-PGKpuro2ABFP-W by cloning in the ccdB resistance gene cassette from pKLV1-fl-U6gRNA(BbsI)-ccdB-PGKpuro2ABFP to generate a modified pKLV2-U6gRNA(BbsI)-ccdB-PGKpuro2ABFP-W vector (Addgene #153033). The library was delivered into electrocompetent cells (Lucigen Endura ElectroCompetent Cells, Lucigen) by electroporation with multiple parallel transformations to maintain library representation, before propagation in LB supplemented with 100 μg/mL ampicillin, shaking at 30°C overnight. *E. coli* were harvested and the plasmids were column purified (Qiagen) before production of lentivirus.

#### Lentivirus

For virus packaging, HEK293T cells were co-transfected with the psPAX2 (Addgene #12260), pMD2.G (Addgene #12259) and library plasmid at a 3:1:5 mass ratio using FuGene HD (Promega) in Opti-MEM (Thermo Fisher). Media was refreshed the next day and viral supernatant was collected 72 h post-transfection, filtered and frozen. For CRISPR-Cas9 screening libraries, thawed viral supernatant titer was assessed by infection of target cells, always in the presence of 8 μg/mL polybrene (Sigma-Aldrich), and 48 h later, measuring BFP expression by flow cytometry.

#### Whole-genome CRISPR-Cas9 KO screens

##### Cell line cytokine CRISPR-Cas9 screening

Cas9 expressing cell lines, and *JAK1* or *JAK2* KO clones thereof, were transduced with the human MinLibCas9 virus (Addgene #164896) in the presence of 8 μg/mL polybrene (Sigma-Aldrich), titrated using a BFP fluorophore to achieve an infection rate of approximately 30% in each cells type, as measured on a BD LSR Fortessa instrument (BD Biosciences). HT-29, A375 and SK-MEL-2 cells were selected with puromycin (3 μg/mL or 2 μg/mL for HT-29, Thermo Fisher) for four days, maintaining a 300 × coverage (estimated cells/gRNA), before taking a time 0 cell pellet. Screens were split into cytokine treatment arms and a control arm then cultured for a further 8 days, with passaging or refreshing cytokine every 3–4 days. Remaining cells were then pelleted and stored at −80°C for DNA extraction. For culture of cell lines during CRISPR-Cas9 KO screening in IFN-γ, IFN-β, and IL-6, we treated cells with pre-optimized doses that reduced the growth rate or viability to ∼50% of parental cells (400 U/mL; Thermo Fisher, 400 U/mL; STEMCELL Technologies, 20 ng/mL; Thermo Fisher, respectively) as measured by CellTiter-Glo (Progema) or Incucyte S3 (Sartorious). IL-6 was the exception as it did not alter cell growth but still affected signaling by Western blot. Each screen was independently repeated twice on separate weeks.

##### Autologous tumoroid T cell co-culture and cytokine CRISPR-Cas9 screening

gRNAs from the minimal genome-wide human CRISPR-Cas9 library (MinLibCas9) were utilized. Tumoroids were dissociated into single cells, and a total of 3.3×10^7^ cells were transduced overnight in suspension with the lentiviral-packaged whole-genome gRNA library. The transduction was performed at 30% efficiency to ensure 200× library coverage, with polybrene (8 μg/mL) included. To ensure high cell yields, tumoroids were cultured in suspension with 5% basement membrane extract (BME) as previously described.^75^ After 48 h, tumoroids underwent puromycin selection (2 mg/mL). Fourteen days later, approximately 2×10^7^ cells were collected, pelleted, and stored at −80°C for DNA extraction. Additional cell pellets were collected at key time points: prior to cytokine or T cell exposure (T0), after cytokine treatment (200 ng/mL IFN-γ or 100 ng/mL TNF-*α*), and following the first and second rounds of T cell-mediated killing. Library transduced tumoroids were then stimulated with IFN-γ or TNF-*α* or left unstimulated as a negative control for 9–10 days. For T cell screens, the killing assay was designed to achieve approximately 50% tumoroid elimination, enabling the identification of genes associated with resistance and sensitization. The effect of T cells was initially titrated in a smaller format and subsequently scaled up to reach the desired killing efficiency. Tumoroids underwent two rounds of T cell exposure, each conducted at an optimized effector-to-target (E:T) ratio of 1:1. T cell selections (killing assays) were conducted across multiple 6-well plates and lasted for 72 h. Depleting nicotinamide from the complete CRC tumoroid medium had only a mild impact on tumoroid growth over a 3-day period and significantly improved their viability over one week compared to culturing in T cell medium. Importantly, nicotinamide depletion did not compromise the T cell killing capacity of CRC-9 tumoroids. Based on these findings, all screens were conducted using a tumoroid medium depleted of nicotinamide to maintain tumoroid viability, while ensuring optimal T cell function. After each selection, cells were reseeded in 5% BME-supplemented media and cultured until reaching a minimum of 4×10^7^ cells, enabling subsequent selections and pellet collection for analysis.

#### Molecular biology cloning

Individual gRNAs sequences were extracted from MinLib gRNAs (Addgene #164896), ordered as oligos (Sigma-Aldrich), and cloned using Golden Gate cloning. Our procedure made use of primers encoding a gRNA with BbsI overhangs and an additional G for hU6 RNApolIII transcription (Forward: 5′-CACCGNNNNNNNNNNNNNNNNNNN-3′ and Reverse: 5′-AAACNNNNNNNNNNNNNNNNNNC-3′), annealed by boiling (100°C for 5 min) and slowly cooling to room temperature (0.1°C per second until reaching 25°C) before ligating duplexes with a BbsI entry vector (Addgene #67974 or a hygromycin-mAzami Green version) using BbsI-HF (NEB), T4 DNA ligase and buffer (NEB), 1× BSA (NEB) for 30× cutting (37°C for 5 min) and ligating (16°C for 10 min) cycles, before heat-shock transformation of Stable Competent *E. coli* (NEB - C3040I) and spreading on 100 μg/mL ampicillin agar plates overnight at 37°C. Colonies were picked and expanded in LB containing 100 μg/mL ampicillin before shaking at 30°C overnight. *E. coli* were harvested and the plasmids were column purified (Qiagen) and sequences were verified via Sanger sequencing of the plasmid with a U6 promoter specific primer (Eurofins). All gRNAs are listed in Table S4.

#### Validation cell lines

Lines were transduced with the gRNA virus supernatant (20% well media volume plus 8 μg/mL polybrene; Thermo Fisher) and selected with antibiotics (puromycin or hygromycin). To produce lines with multiple targeted genes, sequential rounds of infection and antibiotic selection was employed. To mitigate the gene independent effect of multiple infections, a non-targeting guides containing virus was also applied to single KO populations. For confirming *CHD1*, *MAP3K7* and *CDX2* knockout or knockdown and their effect on cytokine signaling, the protein expression of targeted genes together with P-STAT1, cleaved caspase 3, and caspase 8 was assessed by treating cells with IFN-γ (500 U/mL; Thermo Fisher) and/or TNF-*α* (100 ng/mL; Thermo Fisher) for1 h, 8 h, and 24 h before Western blotting. Cell lines with the greatest reduction in targeted protein expression were chosen for downstream experiments. For proliferation and viability assays, cells were plated in white opaque 96 well plates (Corning) in triplicate to allow for endpoint Cell Titer-Glo viability assessment. Cells were treated with 500 U/mL IFN-γ (Thermo Fisher) imaging using Incucyte S3 (Sartorius) every 8 h. In the case of VCaP lines, IFN-γ was refreshed halfway through the culture period given the length of the assay (8 days), all other lines were optimized for a 6-day assay. At the end of the growth periods viability was determined using Cell Teter-Glo. Additionally, given the morphology of this cell line, VCaP cells were unable to be optimized for confluency assessment. For Takinib drug treatments, HT-29 cells were treated with IFN-γ (400 U/mL; Thermo Fisher) in the presence or absence of Takinib (5nM–50μM) for six days following which viability was assessed by Cell Titer-Glo.

#### Validation autologous tumoroid T cell co-culture

Growth and maintenance of CRC-9 tumor organoids in 3D was achieved by growth in 80% basement membrane extract (BME) (R&D Systems). CRC-9 tumoroid T cell co-culture assays were performed as described.^74^ CRC-9 Cas9-mCherry cells with NT gRNA (mAzami Green) were 1:1 co-cultured with cells with KO gRNA (BFP) in a competition assay. Briefly, 96 and 24 well-plates were coated with anti-CD28 antibody (2.2 μg/mL), then PBMCs were thawed and cultured at 2 × 10^6^ cells per well in the 24 well-plate with T cell thawing media (RPMI, 1% penicillin-streptomycin, 1% Glutamax, 10% FBS (Gibco), and 150 U/mL IL-2 (Thermo Fisher)). On the same day, autologous CRC-9 tumoroids were removed from their 5% BME and replated with 0% BME and IFN-γ (Thermo Fisher) at 3 × 10^6^ cells/mL in 2 mL culture media (RPMI, 1% penicillin-streptomycin, 1% Glutamax, (Gibco), and 10% Human Serum (Sigma-Aldrich)). The following day, T cells and tumoroids (5,000 cells of BFP gRNA and 5,000 cells of mAzami Green gRNA) were plated in the prepared 96 well plate in suspension at a 3:1 effector:target (E:T) ratio for 72 h. Plates were placed in an Incucyte S3 (Sartorius) and wells were imaged every 6 h with phase and fluorescent optics for mCherry and mAzami Green. All assays were performed in the presence of the anti-PD-1 antibody nivolumab (20 μg/mL; Selleckchem) in culture media supplemented with primocin (Invivogen).

#### Syngeneic transplantation

We generated two lentiviral gRNA vectors targeting the mouse *Chd1* and *Map3k7* and verified KO and dKO through dual infection by Western blotting following puromycin selection. gRNA 2 for *Map3k7* and *Chd1* were chosen for downstream *in vivo* experiments. As a control cell line, we used B16-F10 cells dual infected with a non-targeting gRNA (NT). Primers used to generate gRNAs are listed in Table S4. 1 × 10^6^ B16-F10 Cas9 cells were subcutaneously injected in 100 μL endotoxin-free PBS into the flank of C57BL/6 mice (Charles River Laboratories). Experiments were blinded and tumors were monitored with calipers every 2–3 days by facility staff. Seven days post-engraftment, mice were treated with 0.2 mg each (i.e., 10 mg/kg for a 20 g mouse) of isotype-matched control antibodies (BP0089, BE02060, BioXCell), or anti-PD1 (RMP1-14) and anti-CTLA-4 (UC10-4F10-11) antibodies (BP0146, BE0032, BioXCell), dosing every 2–3 days for a total of three doses.

#### Tissue preparation

##### Lymph nodes

For analysis of single cell preparations from tumor-draining lymph nodes (tdLNs) and non-draining lymph nodes (ndLNs), inguinal tdLNs were dissected and removed from the tumor if attached and harvested into 500 μL RPMI. Inguinal ndLNs contralateral to the tumor were harvested into 500 μL RPMI (without serum added). Lymph nodes were chopped finely using scissors and tissue was digested with collagenase I (563 U/mL) and DNase I (0.225 mg/mL) in 500 μL total volume of RPMI. Samples were incubated for 30 min at 37°C with agitation, then resuspended to obtain a single cell suspension, and pelleted at 600 g for 5 min at room temperature. Lymph nodes were resuspended in PBS supplemented with 2% heat-inactivated FBS (Gibco). 100 μL of each lymph node sample was plated per well for staining and analysis by flow cytometry.

##### Tumors

For analysis of single cell preparations from B16-F10 subcutaneous tumors, tumors were dissected, weighed and harvested into 1 mL RPMI. We only harvested tumors from mice that were macroscopically visible by dissection at endpoint. Tumors were chopped finely using scissors and decanted into a 15 mL Falcon tube. 3 mL of tissue digestion buffer containing collagenase I (563 U/mL) and DNase I (0.225 mg/mL), made in RPMI was added per tumor sample. Samples were incubated for 45 min at 37°C with agitation. Samples were then strained through a 70 μm filter to obtain a single cell suspension and centrifuged at 1500 rpm for 5 min at 4°C. Red blood cell lysis (Qiagen) was done for 3 min at room temperature, followed by centrifugation and resuspension in 1 mL of PBS supplemented with 2% heat inactivated FBS. 100 μL of each tumor sample was plated per well for staining and analysis by flow cytometry.

#### Immunophenotyping of tissues

For staining, samples were incubated with 50 μL of surface antibody stain master mix containing anti-mouse CD16/32 (Thermo Fisher) to block Fc receptors, for 25 min at 4°C. Samples were then washed with 100 μL of PBS, centrifuged at 1500 rpm for 5 min at 4°C. Using the Foxp3/Transcription Factor Kit (Thermo Fisher), samples were fixed for 20 min at room temperature, washed to remove the fixative and permeabilized. Samples were incubated with intracellular antibodies diluted in the permeabilization buffer and incubated overnight at 4°C. Samples were then washed with centrifugation at 1500 rpm for 5 min at 4°C and resuspended in 300 μL of PBS containing 123 count eBeads (Thermo Fisher) to determine absolute cell number.

Surface antibodies used for identification of lymphoid cell populations were CD45 BV510 (30-F11, Biolegend), CD3e PeCy7 (145-2C11, Thermo Fisher), B220 APC-eFluor 780 (RA3-6B2, Thermo Fisher), CD4 AF700 (GK1.5, eBioscience), CD8a BB700 (53–6.7, BD Biosciences), NK1.1 BUV 395 (PK136, BD Biosciences). Antibodies used to remove contaminating cell types for each flow cytometry panel were labeled “lineage” on flow plots. For lymphoid phenotyping, antibodies were conjugated to eFluor 450 and were FceRIa (MAR-1, eBioscience), CD172α (P84, Biolegend), Siglec-F (1RNM44N, eBioscience), XCR1 (ZET, Biolegend), CD64 (X54-5/7.1, Biolegend), CD11b (M1/70, eBioscience), CD11c (N418, eBioscience), F4/80 (BM8, eBioscience), Ly-6C (HK1.4, eBioscience). Lineage antibodies for myeloid phenotyping were conjugated to eFluor 450 (eBioscience) and were CD3 (145-2C11), NK1.1 (PK136), CD5 (53–7.3), CD19 (1D3) and B220 (RA3-6B2). Surface antibodies used for the identification of myeloid cell populations were FceRIa PerCP-eFluor710 (MAR-1, eBioscience), CD172α AF488 (P84, Biolegend), Siglec-F SB600 (1RNM44N, eBioscience), XCR1 BV650 (ZET, Biolegend), CD64 BV711 (X54-5/7.1, Biolegend), CD11b BV785 (M1/70, Biolegend), I-A/I-E BUV395 (CI2G9, BD), CD11c AF700 (N418, eBioscience), F4/80 APC-eFluor780 (BM8, eBioscience), Ly6G PE-eFluor610 (1A8-Ly6g, eBioscience), Ly-6C PE-Cy7 (HK1.4, eBioscience). The viability dye used for lymphoid and myeloid panels was a fixable viability dye, UV 455 (eBioscience). Data was acquired on a BD Symphony instrument and analyzed using FlowJo Version 10.10.0.

#### RNA preparation

Cells were plated to achieve an approximate 80% confluency at the point of harvest for RNA extraction and cultured for 1 day prior treatment with IFN-γ at 500 U/mL (Thermo Fisher) for 0, 1 and 24 h in one experimental set (HT-29 and VCaP CHD1-MAP3K7) and 0 and 72 h in the second experimental set (HT-29 CHD1-MAP3K7-CDX2). RNA was extracted in wells (RNeasy, Qiagen), DNA removed with DNase I digest (Qiagen) prior to library preparation.

#### Next-generation sequencing

##### CRISPR-Cas9 cell line KO screens

Genomic DNA was extracted from cell pellets (DNeasy Blood and Tissue Kit; Qiagen) and the gRNA cassette was PCR amplified (PCR1: 28 cycles, 0.3 μM each primer, gDNA 3 μg/reaction using KAPA HiFi HotStart Ready Mix; Roche) with 24 reactions in parallel to maintain the complexity of the library. Each PCR1 product was QC checked by running on a gel to confirm that the bands were of similar strengths. Replicates were then pooled (5 μL removed from each and combined) and column purified (QIAquick PCR Purification Kit; Qiagen) before quantification using a Qubit - High Sensitivity kit (dsDNA Quantification Assay Kit; Thermo Fisher) and diluted to 200 pg/μL in nuclease free water.

PCR1 products were then indexed (PCR2: 8 cycles, 0.2 μM each primer, 1 ng PCR1, using KAPA HiFi HotStart Ready Mix; Roche) and purified using AMPure beads (40 μL of beads to 50 μL sample; Beckman Coulter). Each PCR2 product was verified for purity and size using 2100 Bioanalyzer using the Bioanalyzer High Sensitivity DNA Analysis kit (Agilent). Libraries were sequenced on the HiSeq2500 (Illumina) using 19 bp SE sequencing on Rapid Run mode with a custom primer (9385110-U6-Illumina-seq2). All primers and PCR programs used are listed in Table S4.

##### CRISPR-Cas9 CRC-9 organoid KO screens

Genomic DNA was extracted using the Qiagen Blood & Cell Culture DNA Maxi Kit (13362) following the manufacturer’s protocol. PCR amplification, Illumina sequencing (19-bp single-end sequencing with custom primers on the HiSeq2000 v.4 platform.

##### RNA-seq sequencing

RNA was reverse transcribed using poly dT priming (SuperScript IV, Thermo Fisher). Libraries were sequenced on the NovaSeq6000 (Illumina) using 150 bp SP sequencing. All RNA sequencing experiments were repeated independently two-three times on separate days and averaged as indicated in figure legends. All primers are listed in Table S4.

#### Incucyte S3

For all Incucyte S3 (Sartorius) based experiments, except autologous tumoroid-T cell co-cultures, cells were seeded and allowed to adhere for 1 day prior to treatment and imaging commencing, and confluency was determined by phase confluence with classic confluence segmentation on Incucyte S3 software (v2018B). For autologous tumoroid-T cell co-cultures, plates were imaged immediately after commencing the co-culture and mAzami Green and mCherry cell numbers were determined using Top-Hat segmentation. For autologous tumoroid T cell co-culture, T cell killing was quantified by comparing the relative cell numbers between the BFP-gRNA (mCherry only) and NT-mAzami Green-gRNA (mCherry and mAzami Green) to give ratios which were normalized to the 0 h measurements. Analysis was performed using Incucyte S3 software (v2018B).

#### CellTiter-Glo

Where indicated, CellTiter-Glo viability assays (Promega) were performed to assess cytokine response following manufacturer’s instructions. Briefly, cells were plated in white opaque 96 well plates (Corning) in triplicate. Following cytokine/drug treatment, all media was removed and replaced with 50 μL base media to which 25 μL of CellTiter-Glo (Progema) was added. In the case of CRC-9 tumoroids, following treatments Cell Titer-Glo equivalent to 1/2 of the existing well volume was added. Plates were protected from light and incubated at room temperature for 30 min before measuring well fluorescence (Paradigm). CellTiter-Glo fluorescence values were normalized by calculating them as a percentage of the mean average of their control wells.

#### Western blotting

Cells were lysed in sample loading buffer (8% SDS, 20% β-mercaptoethanol, 40% glycerol, 0.01% bromophenol blue, 0.2 M Tris-HCL pH 6.8 or NuPAGE LDS Sample Buffer 4× with 10% β-mercaptoethanol) and boiled (95°C) for 5 min before loading onto a NuPAGE 4–12% Bis-Tris gel (Thermo Fisher). Proteins were transferred to a PVDF membrane (Amersham) in Tris-Glycine Transfer Buffer (Sera Care) before blotting overnight with the following primary antibodies: β-tubulin (#T4026, 1:2,000) from Sigma-Aldrich, or β-actin (#4970, 1:1,000), CHD1 (#4351, 1:1,000), MAP3K7 (#45206, 1:500), STAT1 (#9172, 1:1,000), pSTAT1-Y710 (#7649, 1:1,000), JAK1 (#3344, 1:1,000), JAK2 (#3230, 1:1,000), Cas9 (#14697, 1:1,000), CDX2 (#3977, 1:1,000), vinculin (#13901, 1:1,000), cleaved caspase 3 (#9661, 1:1,000), and caspase 8 (#9746, 1:1,000) from Cell Signaling Technology. Secondary antibodies (anti-mouse and ant-rabbit) were conjugated to horseradish peroxidase (#NXA931V and #NA934V, Amersham, 1:2,000-1:5,000). Membranes were imaged using G-Box iChemi XL (Syngene) or ImageQuant 800 (Amersham) imagers. Ladder used in all blots was the ECL Rainbow Marker - Full range (Sigma-Aldrich). For loading controls β-tubulin, β-actin or vinculin were used as appropriate.

#### Data analysis

##### CRISPR-Cas9 KO screening

To analyze CRISPR-Cas9 screens we used MAGeCK^112^ to generate comparisons between control and cytokine-treated arms. We compared WT, *JAK1* KO, *JAK2* KO and the independent cell clones of each experiment separately. We pooled replicates from two independent screens to generate an average read-count for each gRNA, with the human MinLibCas9 library having two independent high-activity gRNAs per gene. As part of the implementation of MAGeCK, gRNAs were grouped together to give gene-level log2 fold-change values and relevant statistics. Any gRNAs with a count of 0 in the control samples were filtered out of the downstream analysis. We set a *p*-value cutoff of <0.05 to assess significance and an effect size of log2 fold-change of < −0.5 or >0.5 for sensitizing or resistance gene hits, respectively. For tumoroid screens, FDR <0.2 was used as an additional criterion to increase stringency due to higher signal-to-noise. R code used for downstream analysis is available on GitHub (https://github.com/MatthewACoelho/Watterson_etal_CRISPR_analysis). For pathway enrichment of CRISPR-Cas9 hits in cancer cell models, we used g:Profiler.^113^

##### RNA sequencing

Paired-end transcriptome reads were quality filtered and mapped to GRCh38 (Ensembl build 98) using STAR-v2.5.0c^114^ with a standard set of parameters (https://github.com/cancerit/cgpRna). Resulting bam files were processed to get per gene read count data using HTSeq 0.7.2, which was used for downstream analysis. Using the DESeq2 R library,^115^ merged per gene read counts were filtered to remove duplicates and genes which had <20 counts in ≥6 samples before undergoing differential expression and principal component analysis (https://github.com/ABWatterson/DESeq2_All_RNAseq_PCA and https://github.com/MatthewACoelho/Watterson_etal_RNAseq_analysis). Differential expression analysis was conducted by comparing all samples to one another and plotted using -log10 *p*-value vs. Log2 Fold Change. Normalized and log transformed counts generated in the same DESeq2 pipeline were then used for further analyses. Using the decoupleR R library,^82^ pathway and transcription factor activities were determined. For pathway analysis (https://github.com/ABWatterson/decoupleR-Pathway-activation-main), pathway activity was determined using the top 500 genes per pathway in the PROGENy model. For transcription factor analysis (https://github.com/ABWatterson/decoupleR-TF-activation), transcription factor activity was determined using the CollecTRI network.^116^ Gene Set Enrichment Analysis was performed using software from the Broad Institute (GSEA version 4.3.3^117^^,^^118^) using standard parameters with no collapse (https://www.gsea-msigdb.org/gsea/doc/GSEAUserGuideFrame.html) and normalized counts. All gene sets used in the analysis are listed in Table S3 and were obtained through the GSEA molecular signatures database.

##### Publicly available patient data

Studies in a variety of cancer types were selected from clinically available data that met the criteria of having patients profiled by WGS or RNAseq. Clinical data was acquired from cBioPortal^119^^,^^120^^,^^121^ (TCGA, Other studies). Using cBioPortal, studies were queried for deep deletion status of CHD1 and MAP3K7 in all samples. Samples identified to have a deep deletion in one or both genes were counted and then normalized as a percentage of the total number of samples of that cancer type within the study. Criterion for study inclusion in the final figure was <1% deep deletion in either or both genes. Study data used in this analysis are listed in Table S5.

##### Hartwig Medical Foundation data analysis

Patients receiving “Immunotherapy” as their “consolidatedTreatmentType” were selected from the isofox RNA sequencing dataset (464/3,682). Clinical benefit (CB) was determined as a “firstResponse” of “PR” (partial response), “CR” (complete response). No clinical benefit (NCB) was defined as a “firstResponse” of “SD” (stable disease), “PD” (progressive disease), or “Clinical progression”, in line with previous reports.^94^ We grouped cohorts by primary tumor location and filtered on groups >50 patients on ICB; melanoma/skin, urothelial tract and lung. For RNA analysis we used normalized TPM (adjusted transcripts per million) plotted on a log-scale. All data analysis was performed using virtual machines with R and Unix on Google Cloud Platform in accordance with the Data Access Agreement with HMF. The Purple pipeline was used to extract mutations in driver genes, which included CHD1 but not MAP3K7. ICB was anti-PD-1 (*n* = 53), anti-PD-L1 (*n* = 2) or anti-PD-1/CTLA-4 combination (*n* = 1) for lung cancer, and anti-PD-1 (*n* = 100), anti-CTLA-4 (*n* = 6) or anti-PD-1/CTLA-4 combination (*n* = 35) for melanoma. 1/141 melanoma patients treated with ICB had a deletion of CHD1 in their tumor and clinical response. Sample sizes are indicated in the figures.

### Quantification and statistical analysis

Statistical tests, exact value and description of n, definition of center, dispersion and precision measures are described in the figure legends. No randomization was performed and no statistical methods were used for sample size determination. For CRISPR-Cas9 screening analysis with MAGeCK, *p* < 0.05 and a false discovery rate (FDR) of <10% (cell line based screens) or <20% (CRC-9 based screens with higher experimental noise) were used as significance thresholds. For bulk RNA-seq analysis, exclusion criteria were RNAs with low read counts (genes which had <20 counts in ≥6 samples) or replicates with Pearson’s correlation <0.9 and poor clustering by Euclidean distance between sample replicates, meaning we excluded VCap *CHD1* gRNA2 IFN-γ replicate 3 (Figure S6A). For multiple paired *t* test, Two-tailed unpaired Student’s *t* test, two-sided Fisher’s exact test, Wilcoxon signed-rank test, and two-way or one-way analysis of variance (ANOVA), significance was defined as *P* < 0.05.

## Supplemental Information

Document S1. Figures S1–S8

Table S1. Quality control metrics for CRISPR KO screen counts

Table S2. Processed data from CRISPR KO screens

Table S3. RNA sequencing GSEA analysis

Table S4. Primers and gRNA sequences

Table S5. TCGA study sample data

Table S6. DNA and RNA sequencing ENA accession numbers

Table S7. Raw CRISPR read counts

Document S2. Article plus supplemental information
